# A Rare Rheumatologic Case of Catastrophic Antiphospholipid Syndrome

**DOI:** 10.7759/cureus.41972

**Published:** 2023-07-16

**Authors:** Mani Maheshwari, Hemanthkumar Athiraman

**Affiliations:** 1 Hospital Medicine, Banner Health, Mesa, USA

**Keywords:** acute renal failure, autoimmune syndromes, corticosteroid treatment, thrombotic microangiopathy (tma), rituximab therapy, lupus anticoagulant, catastrophic antiphospholipid syndrome (caps), hemophagocytic lymphohistiocytosis (hlh), cmv viremia

## Abstract

A very rare and severe disease catastrophic antiphospholipid syndrome is defined by small vessel occlusions resulting in multi-organ involvement in the presence of antiphospholipid antibodies. This case report presents a case of catastrophic antiphospholipid syndrome in a young female without past medical history.

## Introduction

Catastrophic antiphospholipid syndrome is an autoimmune disease defined by multi-organ involvement by small vessel occlusions and the presence of antiphospholipid antibodies [1]. Catastrophic antiphospholipid syndrome is the most severe form of antiphospholipid syndrome [2]. Less than 1% of patients with antiphospholipid syndrome develop catastrophic antiphospholipid syndrome, and its high mortality rate makes it very significant in the medical field [3]. Approximately 49% of catastrophic antiphospholipid syndrome cases were triggered by infection, 72% were characterized by renal involvement, and 19% reported pathology results with evidence of thrombotic microangiopathy [3]. This is a case presentation of a young female patient with catastrophic antiphospholipid syndrome and the following set of findings: infection, renal involvement, and thrombotic microangiopathy.

## Case presentation

A 32-year-old female without past medical history was sent to the emergency room by her primary care physician after she failed antibiotic treatment for pneumonia. She complained of fevers, night sweats, productive cough with yellow sputum, myalgias, decreased appetite, nausea, and vomiting for the past two weeks. She was negative for coronavirus disease 2019 (COVID-19), respiratory syncytial virus, and influenza in the emergency room. She also complained of dark urine. She had apparent respiratory distress and severe chest pain, which was pressure-like, sharp, and worsened with coughing and movement. Her vital signs were: blood pressure of 126/82 mmHg, heart rate of 134 beats/minute, respiratory rate of 24 breaths/minute, pulse oximetry of 98% on room air, and temperature of 101.0° Fahrenheit. Laboratory assessment on admission is shown in Table 1.

**Table 1:** Laboratory assessment on admission COVID-19: Coronavirus disease 2019

Test	Result	Interpretation
COVID-19	Negative	Negative
Respiratory syncytial virus	Negative	Negative
Influenza	Negative	Negative
Hemoglobin	11.9 g/dL	Normal Range
Hematocrit	35.60%	Normal Range
Platelet	99 K/uL	Low
Blood urea nitrogen	41 mg/dL	High
Creatinine	1.78 mg/dL	High
Sodium	131 mmol/L	Low
Potassium	5.4 mmol/L	High
Albumin	2.5 g/dL	Low
Alkaline Phosphatase	165 U/L	High
INR (international normalized ratio)	1.4	High
Fibrinogen	966 mg/dL	High
D-dimer	>7650 ng/mL	High

**Figure 1:**
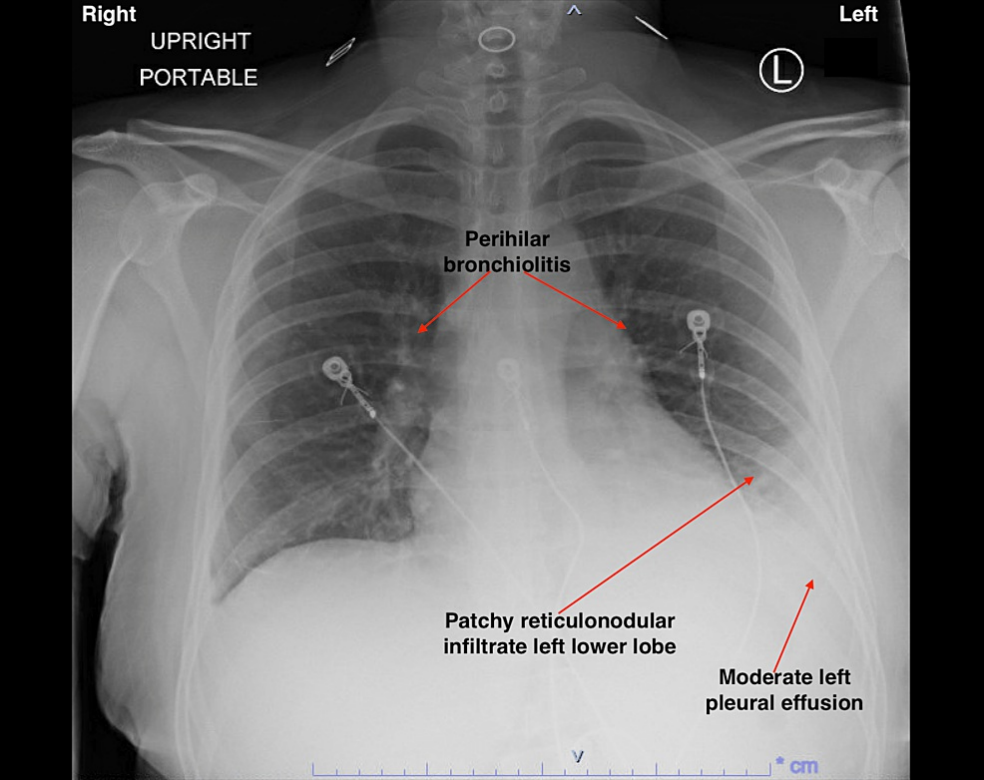
Chest X-ray on admission The image is showing a patchy reticulonodular infiltrate left lower lobe, moderate left pleural effusion, and perihilar bronchiolitis.

Urinalysis revealed: a cloudy appearance, trace ketones, moderate protein, small blood, trace leukocyte esterase, 3-10 red blood cells/high-powered field, granular casts, and hyaline casts of >20/low-powered field. Chest X-ray on admission showed patchy reticulonodular infiltrates in the left lower lobe, moderate left pleural effusion, and perihilar bronchiolitis (Figure 1).

**Figure 2:**
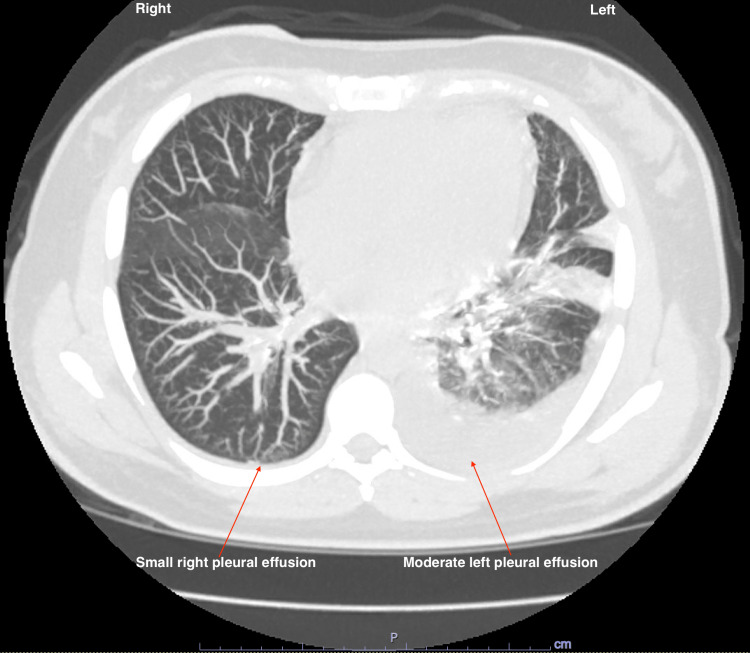
CT chest showing small right and moderate left pleural effusions

CT chest showed small right and moderate left pleural effusions, diffuse bilateral perinephric, and retroperitoneal fat stranding projecting into the bilateral pelvis (Figure 2).

The patient was admitted to the progressive care unit on broad-spectrum antibiotics and underwent left-sided thoracentesis, which revealed 800 mL of yellow fluid (transudate). She clinically deteriorated over the next one to two days, with hypoxia due to reoccurring pleural effusion, developed pancytopenia, and suffered worsening acute kidney injury (creatinine at 4.8 mg/dL), resulting in extensive anasarca, requiring emergent dialysis. She underwent renal biopsy, which showed acute and chronic thrombotic microangiopathy involving the glomeruli and acute tubular injury. Her autoimmune workup results are shown in Table 2.

**Table 2:** Autoimmune workup results

Test	Result	Interpretation
Anti-Nuclear Antibody	Positive	Positive
Anti-Nuclear Antibody Titer	Titer of 1:320	High
Anti-Nuclear Antibody Pattern	Speckled	Indicates Rheumatic Disease
Avise Lupus Test	Positive	Positive
Anti-Sjögren's Syndrome Antibody (Anti-SSA Ab)	Positive	Positive
Anti-SSA 52 Antibody	High	High
Anti-SSA 60 Antibody	>240 u/mL	High
Anti-Thyroglobulin Antibody	78.0 IU/mL	High
BC4d Flowcytometry (Percentage of C4d bound to B lymphocytes)	81	High
EC4d Flowcytometry (Percentage of C4d bound to erythrocytes)	21	High
Dilute Russel Viper Venom Test	1.54 Ratio	High
Normalized Dilute Russel Viper Venom Test	1.35 Ratio	High
Ferritin	1,381 ng/mL	High
ADAMTs13 Activity	48%	Low

**Figure 3:**
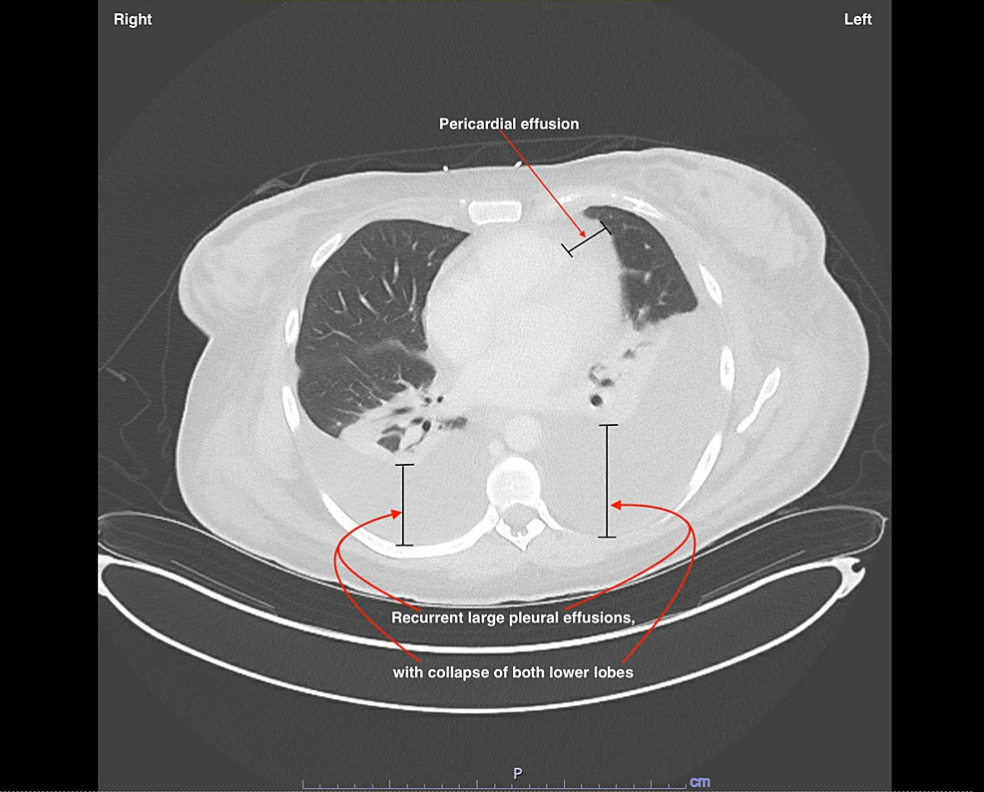
CT chest image The image shows recurrent large pleural effusions with the collapse of both lower lobes and pericardial effusion.

Nuclear Medicine Lung Perfusion Particulate Scan showed subsegmental perfusion defects of the posterior right lower and basilar left lower lobes. A Heparin drip was started due to the possibility of pulmonary emboli. The patient received intravenous (IV) steroids, intravenous immunoglobulin (IVIG), and mycophenolate and was initiated on plasmapheresis. Due to ascites, the patient underwent paracentesis, and fluid analysis was done, which did not indicate spontaneous bacterial peritonitis. Given persistent abdominal distention and worsening hypoxia, the patient underwent a repeat CT chest, which showed extensive third spacing, including anasarca, and large pleural effusions resulting in the collapse of both lower lobes and pericardial effusion (Figure 3). Ascites and a left renal subcapsular hematoma with partial renal infarct were also seen on the CT chest. The patient developed hematuria, and due to continued thrombocytopenia, there was a concern for disseminated intravascular coagulation; hence, the heparin drip was stopped. 

**Figure 4:**
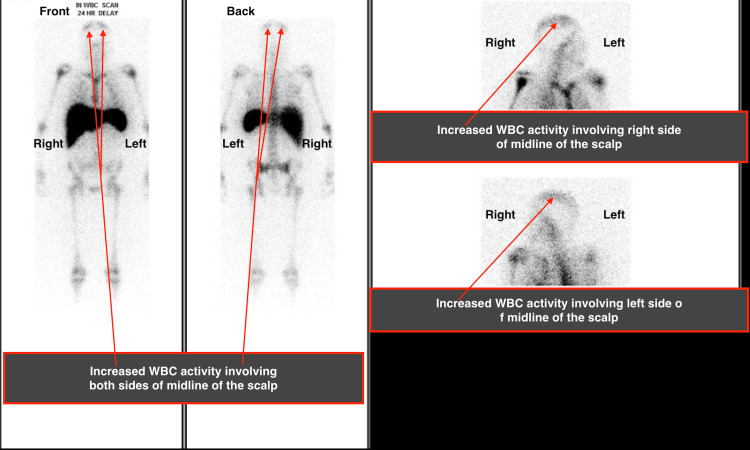
Indium-111 white blood cells imaging study The study showed increased activity involving the scalp on both sides of the midline.

The patient was transferred to a nearby University Hospital for rheumatology evaluation since heparin drip, IV steroids, IVIG, mycophenolate, and plasmapheresis failed to arrest the disease acutely. Complement levels, including C3, C4, and C1q, were low. Catastrophic Antiphospholipid Antibody Syndrome was diagnosed, and the patient was started on rituximab and hydroxychloroquine. The patient continued to be febrile despite negative blood, urine, pleural, peritoneal, and central line tip cultures and despite being on broad-spectrum antibiotics and antifungals. An Indium-111 White Blood Cells Imaging Study showed increased activity involving the scalp on both sides of the midline (Figure 4). Anticoagulation likely alleviated skin manifestations quickly, as the patient only complained of scalp pruritis.

Due to immunosuppressed status, a battery of viral tests revealed cytomegalovirus viremia, and the patient was subsequently started on ganciclovir. She developed worsening pancytopenia; hence ganciclovir was discontinued, and she was started on valganciclovir. Bone marrow biopsy showed normocellular bone marrow (>70%) with a granulocytic shift toward immaturity and markedly decreased granulocytic maturation to the neutrophil stage. The possibility of leukoneutropenia and lymphocytosis from Hemophagocytic Lymphohistocytiosis (HLH) from cytomegalovirus-induced bone marrow suppression arose. Her white blood cell count fell to 1.2 K/mL, and filgrastim was initiated. The patient cleared cytomegalovirus viremia, and antiviral therapy was stopped. She continued to improve and stabilize on rituximab, and she was discharged from the hospital on and close outpatient follow-up visit.

## Discussion

Catastrophic antiphospholipid syndrome is one of the "true" rheumatologic emergencies (other than central nervous system vasculitis, neonatal lupus, macrophage activation syndrome, and kidney-lung syndrome) [4]. Due to the rarity of this disease, more clinical trials are needed to delineate clear treatment guidelines [5].

Diagnosis of antiphospholipid syndrome is made by laboratory confirmation of the presence of antiphospholipid antibodies, including lupus anticoagulant (Dilute Russell Viper Venom Time), small vessel occlusion in at least one organ or tissue, and evidence of organ involvement. Diagnosis of catastrophic antiphospholipid syndrome is made with evidence of all of the above criteria, with three or more organs affected. In the case discussed in this article, the kidneys, lungs, and skin were affected.

About half the cases of catastrophic antiphospholipid syndrome are triggered by infection, which may activate multiple mechanisms, including inhibiting fibrinolysis and anticoagulants, activation of complements, and various cells, ultimately leading to thrombosis and systemic inflammatory response syndrome [6]. Due to this, heparin has the most profound effect on prognosis due to its anticoagulant and anti-inflammatory properties [6]. Corticosteroids help to reduce mortality by acting as an anti-inflammatory, and plasmapheresis appears to help patients with renal involvement (not sure as to exactly why upon literature review) [6]. Hydroxychloroquine is sometimes used in refractory catastrophic antiphospholipid syndrome by lowering the activation of thrombocytes and hence reducing complex attachment to the membrane [6]. The breakthrough treatment of refractory catastrophic antiphospholipid syndrome proves to be rituximab which stops the production of pathogenic IgG autoantibodies by inducing B cell depletion through a negative feedback loop [6].

Diagnosis of catastrophic antiphospholipid syndrome can be histologically supported by acute thrombotic microangiopathy, and differential diagnoses include hemolytic uremic syndrome, thrombotic thrombocytopenic purpura, disseminated intravascular coagulation, and heparin-induced thrombocytopenia [7]. Other triggers of the catastrophic antiphospholipid syndrome can be surgery, trauma, cancer, and medications (including stopping anticoagulants) [8]. 

Unfortunately, the mortality of catastrophic antiphospholipid syndrome is greater than 50% [9]. Patients treated with a combination of anticoagulants, corticosteroids, and plasma exchange had a high recovery rate of 77-78% [10]. In this particular patient, this combination did not arrest the disease acutely, and the patient had to get transferred to a nearby University hospital for consultation with a rheumatologist to start rituximab therapy, which eventually did arrest the disease acutely.

## Conclusions

Making a prompt diagnosis of catastrophic antiphospholipid syndrome and ensuring timely treatment is extremely important, given the high mortality rate with this disease. A multidisciplinary approach and combination therapy are vital; treatment includes anticoagulation, plasmapheresis, IVIG, and immunosuppressive medications.
